# Hypnotic Suggestion in the Practice of Dentistry

**Published:** 1900-07

**Authors:** Walter H. Neall

**Affiliations:** Philadelphia


### International Dental Journal.

Vol. XXI. July, 1900. No. 7.

### Original Communications.[1]

### HYPNOTIC SUGGESTION IN THE PRACTICE OF DENTISTRY.[2]

BY WALTER H. NEALL, D.D.S., PHILADELPHIA.

If a body of individuals, selected for a specific purpose, should isolate itself from the rest of the world, and each person in that body be imbued with the thought of the good and welfare of its members, yet in due course of time one of those persons would stand out pre-eminently, and would so fashion, control, or dominate the doings of the others that he would be recognized as the ruler, the guide of their common destiny.

Executive ability is the argument advanced for his success in this particular instance; the power of argument is another; the gift of speech-making still another. But it is a well-known fact, and it is a matter of recorded history, that men of exceptional executive ability do not control, men who are giants in argument do not lead, and men who could talk by the hour are not the foremost ones of government or the rulers of their fellow-men.

---

[1] The editor and publishers are not responsible for the views of authors of papers published in this department, nor for any claim to novelty, or otherwise, that may be made by them. No papers will be received for this department that have appeared in any other journal published in the country.

[2] Read before the Academy of Stomatology, Philadelphia, February 27, 1900.

To what subtle influence, then, can one man's power over another be ascribed? Fear? That does not enter into the argument. Desire of peace and concord? They play but little part in the question. Personal appearance, then? What weight has this? A man may be repulsive both as to face and figure, he may be careless in attire, and he may not yield obedience to the niceties of polite society, and yet he will attract you, charm you, fascinate you, as it were. You hearken to his words with respect; the peculiar inflection of his voice holds you a close, attentive listener; you follow his actions, his gestures, unconsciously; the flash of his eye thrills you; you laugh when he is merry, you cry when he is sad; in truth, the individual magnetism, the indomitable will-force of such a person, pervades one's brain and sways it as the wind does the rushes.

It is not so much the words, it is not so much the actions, but it is that something, that individual something, emanating from that person and fastening itself upon your innermost soul. Nor is it an evidence of weakness upon the part of the one who yields to the subtle influence of another's power. Man is amenable to impressions; his faculties respond to various sensations. Harmony is conducive of quietness; it is sedative in its nature; whilst discord, on the contrary, frequently produces the most frightful anguish. Certain sights affect one in diverse ways. Even certain odors are potent factors in controlling the feelings of individuals. And thought sways man to the two extremes,—great felicity or abject despair. Man is therefore a creature of sensations or emotions, if you please. And there is no good reason to the contrary that if the emotions control man, man should not control the emotions. To a circumscribed extent he does, covering his own individuality, and by proper study and training he may further extend that power and exercise it over his fellow-beings.

This controlling influence of one individual over another may be aptly illustrated in the case of educational institutions in which the classes come under several instructors. These instructors may be possessed of equal ability as teachers, each be of good address, of even temper, and yet one will get out of his pupils all that is possible, will fashion the better scholar, will command obedience, and will govern without effort.

The secret is that invisible force, that unseeable thread, emanating from the teacher connecting in perfect harmony with the brains of those under his care and guidance; a species of telepathy, as it were, in which the power—the teacher—is strong, stable, and of exceptional vigor, so that every station—the minds of the pupils—receives clear, sharp, and unmistakable impressions.

This sympathetic bond exists where teacher and pupil are face to face, are in actual contact; and yet this mind accord is also noticed when persons are in different localities, and those, sometimes, far removed. The thoughts dwell upon the absent one, and lo! that person suddenly presents himself. This is treated as a coincidence, and yet this so-called "coincidence" may be the result of the will-power of either or both of the individuals concerned.

Take the trite expression, "I feel it in my bones." A premonition that something was about to happen gave rise to its utterance. One is often seized with a nameless dread that disaster or sorrow will be their part before the day is over. This terror cannot be explained or accounted for, and yet it exists and frequently has its realization either in a calamity, a reverse, or news of a distressing or disturbing kind. This might be explained, in part, by stating that pre-existing facts or occurrences may have led up to this apprehension, this anticipation of misfortune or evil report. At any rate, "coming events cast their shadows before," and frequently the brain of man receives impressions or hints of forthcoming happenings of which by no possible combination of circumstances had any prior indications been presented.

This "mind accord," previously referred to, is further exemplified in that, should one enter a dark room, supposed to be empty, and a person be concealed there, that person's presence is conveyed to the other in some mysterious manner; in fact, that unknown presence seems to be felt.

With the knowledge that man does receive brain hints or intimations that something is about to transpire; that he is capable of training his personal will-power so as to make it subservient to his wishes; and, further, that he is enabled, in a measure, to sway the will of his fellow-man; and also when it is taken into consideration how easily mankind, under certain conditions, is prone to yield obedience to concentrated effort and superior brain-force, Hypnotic Suggestion in the Practice of Dentistry is one of interest and importance.

Hypnotism has become a valuable, a necessary adjunct to medicine. The French physicians have proceeded upon well-established lines to prove its worth and efficacy. Their German brethren have investigated wisely and well. In fact, the medical world has begun to realize its claims and to regard it, not as a plaything, an interesting experiment, but rather as a valuable aid in the treatment of certain disorders or conditions. To put it quaintly, hypnotism might now be placed in the list of drugs as an opiate, a soporific, to be used with skill and circumspection.

In this connection the curious custom of the American Indian medicine man, in treating the sick of his tribe, is worthy of consideration. The hideous painted features, the fantastic garb, the discordant monotonous musical instruments, the peculiar droning song and the weird chanting of the sick one's relatives, are calculated to lull, to soothe the patient. That is the object of the treatment. And this being continued without cessation hour after hour, the sick one is really hypnotized to a certain extent. The medicine man's cure is more in the nature of hypnotic influence than of roots and herbs, although they play their part.

True hypnotism is a form of somnambulism artificially produced, in which the subject's every thought or action becomes subordinate to the will of the operator. In hypnotic suggestion the conditions are not carried to any such decisive point, the first stages simply being employed,—that is, quietness, amenability, and trust.

In this wonderful age, progression impels the dentist to treat the mental side of his patient, so far as controlling and suggesting goes, as well as the physical ailments to which his teeth may be subject, and this necessarily leads the dental practitioner into quite a new phase of dental ministration. A dentist of tact may succeed with his patients, but a dentist of tact, with the aid of hypnotic suggestion, will undoubtedly bring about the greater number of cures and successful operations. A positive man, though he be a skilful dentist, will not succeed when one considers the average; not unless back of his dogmatic nature he has the necessary amount of mind-strength or will-power to control his patients, thus enabling him to carry his ideas to a happy issue. Brute force wins but once, and the dentist without sufficient brain acumen to realize that something else than mere cold-blooded cutting, excavating, and plugging is looked for by a suffering public, will be speedily relegated to a limited circle of patients possessing steel nerves and cast-iron natures.

A dentist has a far loftier aim than to be simply an expert in handling the drill, bur, or filling-instrument. The field for research is large and is ready of exploration for him, and to his allotted work another course of careful study is presented. Not that the dentist should be an expert hypnotist, but rather that his mental force or caliber should be of a timbre calculated to influence, soothe, direct, and in a measure control; trained to produce such effects, if need be. Nor is it expected that live pulps can be removed without the patient experiencing distress, or an abscess be opened without pain, or a tooth be extracted minus the usual suffering, although, if the practice be carried far enough, these could be successfully accomplished.

The idea, briefly, is that the patient's thoughts and actions should be controlled to a certain point, rendering him obedient to the dentist's wishes, so that operations, from which one would shrink under ordinary circumstances, can be endured with fortitude and composure. This delectable condition may not be attributed, by the unthinking, to hypnotic influence or suggestion, but rather to the personal will-power of the operator. What is the personal will-power, then, of the operator? The will, first, is the self-consciousness of ability or strength to control or direct; the knowledge of mind-force greater than that upon which it is directed or exerted; secondly, it may be the realization of a power that is governing or guiding. The dentist, therefore, representing the first definition, is to exert his will-power, and in so doing he necessarily practises the rudimentary principles of hypnotism, and the patient,—definition second,—recognizing a superior force or becoming subjective to the quieting influence of mind concentration, yields ready obedience.

In this concentration of mind-force there should be no hesitation or wavering, even for a moment. If intermitting, it is simply time spent for naught. It is the quiet, authoritative, self-reliant manner that controls, not the boisterous, nervously excitable, or hesitating.

If the patient has lack of confidence in the dentist's ability to cut, drill, excavate, and fill without producing pain, and is fearful to a distressing degree, the first step would be to remove that want of trust, after which comes the subjugation of the will. The latter is analogous, in a measure, to the efforts of a physician in administering an anæsthetic. There is some rebellion at first, but gradually the patient succumbs, and eventually a passive, pliable subject is the result.

This hypnotic force or undue strength of the will may be natural or it may be acquired. If natural in its intensity, then the possessor is endowed with great power for good or evil; if dormant, it may be stimulated; if latent, it may be cultivated, to be directed into one of the two great channels enumerated.

A patient suffers more in anticipation, oft-times, than he does when actually seated in the dentist's chair. Instances of positive illness have been noted, superinduced by the very thought of a dental operation, and that of the most trivial character. When a patient of pronounced nervous idiosyncrasy presents himself, a course of preparatory treatment should be employed by the dental practitioner; not looking towards performing an immediate operation, but rather to gain an ascendency or mastery over the other's will-power. Such a course would prolong the dental operation, no doubt, but it would invariably place the patient in a better condition pending the arrival of the crucial period. This controlling force or influence cannot be produced by hard, severe, or exhaustive manual toil: the day laborer is a fitting example. Neither do the works of fiction possess it, although the individuality of certain writers may dominate their books and, to a certain extent, lend dramatic force to their literary efforts.

The planning of an intricate machine, or of a colossal piece of architecture, and the execution of the same exert an influence only of admiration or praise. The newspapers present public sentiment and comment, and offer discussions pro and con, but they do not compel or force one to believe or even accept. The dentist at his chair, being an educated man, a close observer of human nature, and brought into such close relations with his patients, has an exceptional opportunity of producing or causing this sedative influence. Ofttimes the accomplishment of it is exhaustive. After operating upon a particularly stubborn patient, he will be utterly prostrated. This prostration will not be of the body, the fingers, or the hands, but of the *mind*. The nervous force, the vital energy, seems almost used up, and yet the body be without fatigue. In this instance the resisting power of the patient has not been conquered; the dentist has not proceeded upon the right lines. It is will against will, with a pitched battle as the result, an unsatisfactory operation, and quite probably a dissatisfied patient. The moment the patient takes the chair the full influence of the dentist's governing power must be exerted; he must be master of the situation; resistance must be overcome and passiveness secured. If the conditions are not favorable at one sitting, then the postponement of the operation is indicated until they are.

Man's controlling ability may, very properly, be divided into four distinct classes:

*Class 1.—Simple suggestion* alone, without the coercion of will force or influence, one's credulity being worked upon without special effort of the operator, mere intimation and action being employed.

*Class 2.—Collective suggestive influence,* wherein the combined wills of a number of individuals, acting in concert, influence the will-power of a single one. It partakes, in a measure, of the previous class, only being carried farther in its performance.

*Class 3.—Hypnotic suggestion,* in which a person is influenced and controlled by the exertion of another's will-power *without* being placed in an hypnotic sleep.

*Class 4.—True hypnotism,* the subject coming wholly under the control of the operator and having no other thought than to do his bidding. In this class the subject has passed into the artificial slumber known as hypnotic sleep.

In the practice of dentistry, Class 2, *collective suggestive influence,* necessarily does not have a place, and Class 4, *true hypnotism,* with but few exceptions, has not been employed, although examples in each class make interesting studies. In Class 1 it will be observed that the imagination has much to do with the control of the subject's feelings and actions.

*Example 1.*—A woman of color presented herself at a dentist's office for tooth extraction. She insisted upon having some numbing fluid placed around the affected tooth, a molar. The operator, at that moment being without any such drug, substituted *cold water,* and, after plentifully rubbing it upon the gums, extracted the tooth. The patient insisted that she felt no pain, and that in the future all her teeth should be extracted by the same means.

*Example 2.*—An Irish woman visited a certain dental college for the purpose of having a carious tooth removed. She would not submit to the extraction unless gas was administered. The demonstrator in charge of that particular branch having departed for the day, a mischievous student suggested to his companions, unknown to the patient, a novel mode of performing the operation. He produced a foot-bellows with a long rubber hose attached, and, inserting an end of the latter into the patient's mouth, proceeded to fill her full of air. When the woman was almost on the point of strangulation the tooth was removed, *entirely without pain,* as she declared.

*Example 3.*—A gentleman, a man of learning, wished the extraction of a bothersome tooth by the aid of the chloride of ethyl spray, its working and effect having been explained to him. The dentist proceeded to use the same, when, to his consternation, he discovered that the tube would not spray at all, a few drops of the material merely falling upon the tongue. However, as the gentleman had opened his mouth so wide, and seemed so confident, and the tongue had received an impression that a paralyzing agent was being employed, the dentist decided to extract the tooth and explain the non-working of the spray afterwards. The tooth was removed, and, to the operator's surprise, the patient positively assured him that, "That was the easiest operation under which I ever sat; the chloride of ethyl spray is truly a wonderful product of modern dentistry." Needless to state that, to this day, the patient's mind has not been disabused of the part the imagination played in that particular tooth extraction.

*Example 4.*—A patient of unsound mind, accompanied by his attendant, complained of a hard substance being wedged in the crown of a certain tooth. A minute examination was made and nothing discovered, excepting that he possessed an unusually good set of teeth. The patient was not satisfied, and still persisted that a stone was lodged in one of his molars. The cue was sufficient. The operator sought his laboratory, and, securing a pebble such as is found in the sea-sand, another examination was made and the small stone eventually brought to light. Result, a pleased and relieved patient.

*Example 5.*—A patient presented herself to have an artificial plate eased. Whilst talking, the operator had merely wiped off the plate and then reinserted it, in order to ascertain the exact spot of undue pressure. The patient expressed her satisfaction at the immediate comfort given, and departed satisfied.

*Example 6.*—This also relates to an artificial denture. It had been complained of as being uncomfortable as well as cumbersome. In the dentist's estimation, it was an excellent fit and as light as it was possible to be made with strength and stability. He retired for a few minutes to his laboratory. The sounds of filing a piece of vulcanite and the grinding of a porcelain cup reached the ears of the patient. On the plate being reinserted, the fit was accurate and the cumbersomeness had disappeared. *The plate had not been touched with file, scraper, or wheel in any particular.*

The foregoing examples relate to simple suggestive action, and *not* to the true influence of a stronger will. But they serve to show how easily one's reasoning power is disposed to receive, adapt, or adopt, if put into proper frame of mind for doing so. It is the first step leading to the true hypnosis,—the preparation of the mind, the directing of the thoughts into a certain channel, and the leading off from the thing at heart.

In Class 2 direct and concentrated means are employed. Example 1 presents a case wherein no previous studied attempt had been made to accomplish the result.

*Example 1.*—Subject, male, aged thirty; temperament, nervous; health, good. Mr. T. was one of thirty clerks employed in a large office in Philadelphia. Before his arrival, one morning, a fellow-clerk conceived the idea of a systematic remarking upon Mr. T.'s sickly appearance. When Mr. T. presented himself the plan was carried out, and each clerk had a quiet word of regret or commiseration for the victim. At twelve o'clock he went home ill, and spent several days in bed.

The remaining examples in this class relate to united and specially prepared effort. The manner of proceeding is easy of execution. A subject is selected at random and instructed to concentrate his mind wholly upon the success of the experiment. He is not to combat the will of the operator, nor is he to resist in any particular; he must be passive within the strict meaning of the word. He is then requested to retire from the room, and the audience is directed to conceal an article which he may be *willed* to find. Strict quietness is enjoined, with the further injunction that each person concentrate his or her mind upon the object hidden. The subject, blindfolded, is again brought into the room. The operator stands behind this person and places his thumbs at the base of his skull, with the fingers extending to and upon the temples; thus one line of mind communication has been established. The subject is directed to think of finding a certain secreted article, and to be governed entirely by the influence of his *mind impulses;* that is, when thus blindfolded and held, amid deep silence and intently thinking, after a few moments one seems to have an inordinate desire to pitch forward or to either side. Immediately the impulse is felt, the subject must move in that direction. After this comes a period of rest, and then the impulse is experienced again, and thus intermittingly continued until the concealed object is reached and brought to light. The operator gives no intimation of direction whatever. He simply presses his fingers and thumbs firmly upon the subject's head, keeps his thoughts upon the object to be found, his eyes riveted in that direction, and intensely *wills* that it *must* be discovered.

*Example 1.*—Mrs. S., aged thirty-eight; easy-going, pleasant nature; readily influenced by conditions. She proved an apt subject, and gave herself up entirely to the experiment. Result, hidden watches, handkerchiefs, and minor objects were quickly located and disclosed.

*Example 2.*—Mr. A., aged thirty; a broker; a doubter and a scoffer. In manner, brusque and of quick temper. He requested the experiment tried upon himself. He was accommodated only after he had promised to obey, strictly, the conditions exacted. He made two zigzag trips around the room and finally came to a standstill, with spasmodic twitchings, *upward,* of his head. Finally he pulled the bandage away from his eyes, declaring that "the whole thing was nonsensical." He was now in the middle of the room, having started from one end, directly under a large chandelier. He was requested to look above his head, and there, suspended from the central portion of the gas-fixture, was a handkerchief that he was requested to find. If he had obeyed the *mind impulse,* and had stretched his hand above him, undoubtedly he would have grasped the handkerchief.

In Class 3 four examples are presented, each of recent date.

*Example 1.*—Mrs. W., aged about forty years; of meagre physique, highly nervous, and of fluctuating will-force. She wanted six loose teeth extracted, but positively declared that anæsthetics would not be tolerated. In fact, she had decided that she would suffer until the offending members dropped out. The manner of procedure was as follows:

The operator placed himself in front of the patient and, intently gazing into her eyes, quietly suggested that the teeth could be removed readily and without hurt. She demurred and dropped her eyes. She was commanded to look the operator in the face, and again the intense, unwavering gaze was employed. The hands were passed several times across the patient's forehead, and again the suggestion was made that the teeth could be quickly removed without the least suffering on her part. There was slight rebellion. The operator was determined that she *must* submit. On the assistant handing out the extracting forceps, the teeth speedily reposed in the napkin. She has since declared that she never knew how she ever had the courage to have those objectionable teeth out.

*Example 2.*—Master H., aged six; wilful and peevish. He came with his mother and nurse. Several teeth required filling. He would not open his mouth, was impudent to his parent, and repeatedly struck at the nurse. The mother was despatched on a shopping tour, and the nurse sent into the reception-room. Alone with Master H., the operator stood some distance from the patient and exercised the fixed gaze of the eye. Not a word was said for several moments. Then the lad was informed, in a decided manner, that the teeth were to be filled, and would be filled; that it was folly for him to struggle, and that he *must* obey the dentist's every wish. After repeated strokings of his forehead, and reassuring remarks, never once relaxing the intense gaze, the operation was begun and completed ere the return of the mother.

The boy was not scared or awed; he simply *knew* that he had to submit, and did so.

*Example 3.*—Miss S., not yet out of her teens. Teeth very sensitive, and she correspondingly nervous over the fact. It was impossible for her to sit still; the slightest touch caused her to crouch down in the chair, as well as to jerk her head away from the operator's hands. The same means were employed as in Example 1, only they were carried to a greater extent. She was directed to gaze intently at a bright disk of metal for a considerable time; then the eye concentration, after which the passes over the eyes, and the positive, decided manner of speaking. The result amply paid for the interval spent in preparing her for the ordeal of teeth filling.

*Example 4.*—Mr. R., aged probably fifty; a sallow, highly strung individual, gifted with the fault-finding propensity to a great degree. The sensitiveness of his teeth made this failing all the more glaring. After considerable exertion on the part of the operator, Mr. R. was subdued and became a passive, well-behaved patient, the prescribed lines, quoted in the previous examples, being followed out. At first he objected to the fixed gaze, but finally, at its being persisted in, he relapsed into silence and endured treatment from which he had frequently rebelled, and, on several occasions, deferred until another period.

In Class 4 three examples are given, showing how this class might be employed in operations of a minor character.

*Example 1.*—On several occasions, in a public hall, a master of the art of hypnotism placed a number of individuals in an hypnotic sleep, and whilst in that condition carious teeth were extracted by a dental practitioner, the patients being, to all intents and purposes, oblivious of the whole proceeding. In fact, not a moment of hesitation was noticed. The persons submitted to the extraction quietly and willingly, being under the impression that they were to enjoy sensations of a novel and pleasing character.

*Example 2.*—A young man of average intelligence, being placed in an hypnotic sleep by an experimenter in one of the institutions of learning in this city, pins, needles, etc., were used upon him without a tremor of his muscles. Afterwards he was informed that but one side of his body would be insensible to pain, whilst the other would remain normal. The idea was carried out. The young man felt no distress when needles, pins, and sharp-pointed knife-blades were thrust into his flesh upon the side purported to be senseless to pain, but invariably flinched and drew himself away when the normal side was even touched with the pricking instruments. He was put through other interesting tests, with happy results; that is, the operator's success with this subject was conclusive.

*Example 3.*—Miss W., aged eighteen; pupil of a seminary for young ladies; nervous temperament; bright, active, and vivacious; one of the guests at a summer boarding-house at which the writer was stopping. In this case several experiments in Class 2, collective suggestive influence, had been attempted, and were invariably highly successful, such as finding a handkerchief, a ring, a key, or picking out a certain person from the midst of the audience, some thirty in number. Evening after evening these trials were made with Miss W. as the particular subject. Finally it occurred to the writer, who was conducting the experiments, that if the young lady was so amenable to mind influence, why not carry the attempt to the true hypnotic period. This was proposed and explained to her, and with her consent the test was made, and from that time Miss W. was able to accomplish many astonishing feats at the will of the operator. She was an exceedingly impressionable subject.

For the benefit of the uninitiated, the *modus operandi* is given

*in extenso.* Miss W. was conducted to another apartment, usually by a committee of two. One person was generally selected by the audience and told to write any command or wish upon a piece of paper, said writing afterwards being seen only by the operator. The paper was then concealed. Absolute quietness was now requested, and Miss W. was brought back to the room. The operator looked her fixedly in the eyes and passed his hands lightly once or twice across her forehead. She was then blindfolded. The operator, with his fingers upon the pulse of her right wrist, said, impressively and authoritatively, "A certain thing is required of you to do. I am thinking of that thing. Go and do it."

With that she was released. There would be hesitation for a moment, but, being urged by the sharp command, "Do my bidding!" she would begin a rather uncertain tour of the room, amid profound silence, and when wavering in her movements, a quick injunction reassured her. At the completion of the experiments, the concealed paper always testified to the success of them.

Whilst they produced astonishment and entertainment for the audience, they seemed to afford the young lady in question a peculiar delight, for she invariably sought out the writer "to practise upon her," as she called it, whenever her friends made an evening visit. When asked what her sensations were, she replied, "None worth speaking of. I simply allow my mind to become a blank, if that is possible, and think of nothing but what I must do for Dr. Neall. I seem to be in a dazed condition, being vaguely compelled to do something about which I have a confused idea. After I am told to 'wake up,' I always have this nervous spell. I really do not mind it; it is a little exhausting, but the effects do not last long."

It would be absurd to declare that *every* person could be brought to the stage exemplified in Class 4. It would be simply impossible. But it is without fear of contradiction that the statement is made that almost every person can be brought to feel the influence of Class 3.

Class 1 is employed as a matter of course, often unconsciously so by the dentist, but when Class 3 is used, it is the result of intelligent study. A few hints as to practical work are apropos.

The subject being placed in an easy, comfortable position, a bright object—a highly polished metal disk, set in a rim of rubber, or a new silver coin—is held within eight or twelve inches of the eyes. The gaze is concentrated upon this bright object for from fifteen to twenty minutes, with the mind dwelling only upon this one idea. At the expiration of the time an impressionable person will be in a fit condition to render ready obedience to an authoritative tone. Or, as has been suggested and used in France, several bright objects may be hung upon the ends of fine silken cords, and the whole made to swiftly revolve by aid of special mechanism. The subject keeps his eyes upon the scintillating circle for a given period, and a quiescent, obedient mind results. A number of persons have been so controlled at one time.

*A Simple Lesson in Mind Concentration.*—The learner, or beginner, is to fix his whole thought and attention upon the *back of the head* of a person *unconscious* of the other's presence. The opportunity for such an experiment is afforded at lecture, entertainment, or other public gathering. The novice fixes his earnest gaze upon the back of the head of a friend some seats in advance. He is to determine, to will, that the friend *must* be influenced, and *must* turn around and recognize him. By constant practice this becomes easy of accomplishment. In fact, being aware that some one has his *eyes upon you* is no new thought, but to systematically exercise one's brain in order to produce the effect may be new to many.

The next lesson deals simply with the idea of looking intently into the eyes of a person when talking, steadily, unwaveringly. The power of holding the eye without movement must be practised assiduously. Looking fixedly into one's own eyes in a looking-glass affords excellent opportunity for experiment.

After these lessons have been thoroughly mastered, the graver considerations of the other phases may be entered upon. The line of study for the dentist, in connection with hypnotic suggestion, is in the cultivation of a firm, clear gaze of the eye; an authoritative, positive way of expressing one's self; a determination to overcome resistance; a steady grasp upon one's temper; certain passes of the hand, which in some cases are indispensable; and the gaining of the subject's full confidence.

If these rules are well considered and carried into effect at the chair, the dentist will meet with greater success in difficult, trying operations than if they were ignored or regarded as being of no moment in the practice of dentistry.

